# Demographic, Socioeconomic, and Clinical Determinants of Repeat Suicide Attempts: A Retrospective Study of Emergency Department Visits at a Community Hospital

**DOI:** 10.7759/cureus.94712

**Published:** 2025-10-16

**Authors:** Paul A Scalzo, Nicole Ann E Villa, Kristina Y Shum, Ashini A Patel, Stacy Chou, Madhulika Badri, Evan T Woods, Charles Wen, Allison Atkinson, Amy Hsu, Eduardo D Espiridion

**Affiliations:** 1 Psychiatry, Drexel University College of Medicine, Philadelphia, USA; 2 Psychiatry, Drexel University College of Medicine, Philadelphia , USA

**Keywords:** community hospital, demographic factors, emergency department, repeat self-harm, repeat suicide attempts, suicide

## Abstract

Background: Suicide is a critical public health issue in the United States, yet research is lacking on understanding the population that visits the Emergency Department (ED) for repeated suicide attempts (SA) and self-harm (SH).

Aims: Our aims are to identify and describe the population that revisits the ED for SA/SH, and to determine the factors that influence revisit rates.

Methods: This retrospective study was conducted from August 2022 to April 2024, reviewing ED visits from January 2019 to December 2021 at a single center. Data on demographics, diagnoses, family history, stressors, and outpatient care were analyzed. Statistical analyses, including Kruskal-Wallis, Mann-Whitney U, and correlation, were conducted.

Results: Among 138 participants (mean age 34.47 years), 83 (60.14%) were female, 86 (62.32%) were White, and 77 (55.80%) were on Medicaid. Psychiatric illness was present in 118 (85.51%), with depressive disorders most common, 76 (64.41%). Almost half of participants, 64 (46.38%), had a family psychiatric history, 28 (20.29%) participants had a diagnosis of hypertension and they had a significantly higher average revisit rate (p = 0.049), and 64 (46.38%) participants used a substance during SA/SH, with alcohol being the most frequently used. Oral medication was the most common method for SA, 145 (54.92% of SAs). Prior to the study period, 43 participants (31.16%) had seen a PCP, and only 70 ED visits (20.23%) resulted in scheduled outpatient follow-up. No significant differences were found between revisits and sex, age, payer status, or income (P > 0.05).

Conclusions: Our study demonstrated that psychiatric illness, substance use, and a family history of psychiatric disorders were highly prevalent among individuals with repeat ED visits for SA/SH. We propose that improving scheduled follow-up care after an event, encouraging PCP suicide-risk assessment, and medication management could serve as key intervention areas to reduce reattempts. Future research should focus on targeted interventions for high-risk populations, such as those with chronic diseases such as hypertension.

## Introduction

Suicide is a leading cause of death in the United States, responsible for 49,476 deaths in 2022, a 2.7% increase from 2021 [1]. The burden on the healthcare system is substantial. From 2011 to 2012 and 2019 to 2020, emergency department (ED) visits per capita for suicide attempts (SA) or self-harm (SH) increased annually by an average of 18.8% from 261 to 871 visits per 100,000 individuals [2]. Furthermore, SH with or without suicidal intent is associated with high hospitalization rates [3]. 

Given the rising incidence of suicide and its significant healthcare burden, understanding factors of SA/SH risk is paramount. Suicide is influenced by medical, emotional, psychiatric, and sociodemographic factors, such as psychiatric illness and financial stress [4]. Though nonfatal SH data are not consistently tracked, research shows strong correlations between SH and mental health disorders [5]. Additionally, an SH history increases the risk of subsequent suicide [5]. 

One of the strongest predictors of future SAs is the history of previous attempts. One study found 60% of re-attempts occurred within six months after an initial attempt, and 18% of re-attempts happened within the first month [6]. Those who attempt suicide multiple times often are younger, female, unemployed, unmarried, or have chronic illnesses, family SA history, or a lack of social support [7]. 

Research on repeated attempters often focuses on specific subgroups, such as those with bipolar disorder or substance use disorder (SUD), leaving gaps in understanding the patterns of SA/SH, particularly in relation to outpatient care, psychiatric comorbidities, and stressors. Furthermore, prior research is limited in examining SH and SA together. 

This study aims to address gaps by focusing on people with repeat ED presentations for SA/SH (without suicidal intent) from 2019 to 2021. We seek to identify demographic, socioeconomic, and clinical characteristics of these individuals, and to uncover risk and protective factors that could influence repeated ED visits for SA/SH, with the goal of informing public health strategies. 

## Materials and methods

This IRB-approved, single-center, retrospective study was conducted at Reading Hospital, a community hospital that is part of the Tower Health system in Pennsylvania, USA, from August 2022 to April 2024. The study was exempt from informed consent as it involved analysis of existing data without human subject interaction or intervention. Data was extracted by chart review of electronic medical records within our healthcare system. The chart reviews were conducted by all the study authors only in the hospital. The study sample participants were first identified using the International Classification of Diseases, 10th Revision (ICD-10) codes of suicide attempt or self-harm for people who revisited the ED between 2019 and 2021 after an initial visit for SA/SH [8]. An event was defined as an episode of SA/SH between 2019 and 2021. The number of revisits was calculated by subtracting one from the total number of events for individuals with no pre-2019 events, while the total number of events during the study period was used as the revisits value for those with pre-2019 events. Multiple revisits of the same patient were treated independently.

Variables including demographics, medical and psychiatric history, family psychiatric history, and payer status were collected from the participants’ medical records at the time of their first presentation for an event within our study period (2019 to 2021). Other collected variables for each event include acute or chronic stressors, payer status, chief complaints, discharge dispositions, and outpatient primary and psychiatric provider visits. Columbia-Suicide Severity Rating Scale was collected for visits that had a score [9]. 

Stressors identified before and during the SA/SH event were categorized as acute or chronic, then further categorized as social, psychological, substance-related, financial, legal, or medical. Acute stressors lasted less than three months, except for acute psychological stressors, which were defined as psychiatric symptoms that were present for less than one year. Chronic stressors lasted ≥3 months, except for psychological stressors, which were defined as symptoms that persisted for ≥1 year. Income levels were categorized by median household income at participants' zip codes from the 2022 American Community Survey 5-Year Estimates (US Census Bureau): low (<50% of the state median), moderate (≥50% and <80%), middle (≥80% and <120%), and high income (≥120%) [10]. 

Mann-Whitney U test, Kruskal-Wallis H test, Spearman Correlation Coefficient, Regression Analysis, and ANOVA were performed when appropriate. The level of significance for these tests was set at p < 0.05 (less than 0.05). All statistical analyses were performed using Python version 3.8.10 with statistical libraries NumPy, SciPy, and pandas [11-13].

## Results

Participant demographics 

A total of 138 participants met the inclusion criteria. The mean age was 34.47 years, and the largest age group was between 35 and 54 years old (n=56; 40.58%). In this sample, 83 (60.14%) were female, 86 (62.32%) were White, 97 (70.29%) were non-Hispanic or Latino, and 77 (55.80%) were insured by Medicaid. Nearly half of the participants, 60 (43.48%), were classified as middle-income (Table 1). 

**Table 1:** Demographics of Study Population This table summarizes the demographic characteristics of the study population, first including mean age and standard deviation (SD), median age, range of ages. It also includes the frequency and percentage of those in different age groups, sexes, races, ethnicities, payer statuses, and income levels. For each group, the average revisit number with standard deviation is listed (- is used in this column for the age summary section of this table that is not a group). Kruskal–Wallis H test p-values are reported for differences in the distribution of revisits between groups defined by each characteristic. *Post-hoc pairwise comparisons by Mann-Whitney U test with Bonferroni correction revealed no statistically significant group differences.

Demographic Characteristics (N = 138)		Average Revisit ± Standard Deviation	p-value
Age, years			-
Mean (SD)	34.47 (15.96)	-	
Median	31.5	-	
Range (max-min)	77 (81-4)	-	
Age Group, number of individuals (%)			0.401
0-17	25 (18.12)	2.32 ± 1.41	-
18-34	37 (26.81)	2.00 ± 1.56	
35-54	56 (40.58)	2.25 ± 1.31	
55+	20 (14.49)	2.65 ± 1.93	
Sex, number of individuals (%)			0.846
Female	83 (60.14)	2.31 ± 1.61	-
Male	55 (39.86)	2.16 ± 1.32	
Race, number of individuals (%)			0.017*
White	86 (62.32)	2.40 ± 1.42	-
Other	38 (27.54)	1.92 ± 1.62	
Black or African American	11 (7.97)	2.00 ± 1.61	
Unspecified	3 (2.17)	3.33 ± 0.58	
Ethnicity, number of individuals (%)			0.190
Not Hispanic or Latino	97 (70.29)	2.39 ± 1.60	-
Hispanic or Latino	40 (28.99)	1.90 ± 1.15	
Unspecified	1 (0.72)	3.00 ± 0.00	
Payer, number of individuals (%)			0.354
Medicaid	77 (55.80)	2.29 ± 1.60	-
Private Insurance	30 (21.74)	1.97 ± 1.07	
Medicare	25 (18.12)	2.56 ± 1.50	
Self-Pay	4 (2.90)	2.50 ± 2.38	
Other Category	2 (1.45)	1.00 ± 0.00	
Income Level, number of individuals (%)			0.076
Middle Income	60 (43.48)	2.13 ± 1.31	-
Moderate Income	31 (22.46)	2.97 ± 2.02	
High Income	28 (20.29)	2.04 ± 1.10	
Low Income	19 (13.77)	1.79 ± 1.23	

Revisit rates by demographics and clinical characteristics 

Using the Kruskal-Wallis H test, there were no statistically significant differences in the distribution of revisit rates by sex (p = 0.846), age group (p = 0.401), insurance status (p = 0.354), income level (p = 0.076), or ethnicity (p = 0.190). Although there was an overall significant difference in revisit rates by race (p = 0.017), post-hoc pairwise comparisons using the Mann-Whitney U test with Bonferroni correction revealed no statistically significant group-level differences (Table 1). 

Psychiatric diagnoses were present in 118 (85.51%) participants, with most individuals having more than two conditions. The average number of revisits rose with the number of psychiatric diagnoses, from 1.8 in those without any diagnosis to 2.77 in those with four or more, though this trend did not reach statistical significance (p = 0.082) (Tables 2-3).

**Table 2:** Psychiatric Disorder Categories/Diagnoses This table presents the prevalence of psychiatric diagnoses among the 118 participants who were documented as having at least one psychiatric disorder. It lists the Diagnostic and Statistical Manual of Mental Disorders, Fifth Edition, Text Revision (DSMV-TR) psychiatric disorder categories and specific diagnoses when given in the chart, highlighting the proportion of the total sample diagnosed within each section [14]. Diagnoses are not mutually exclusive, meaning individuals could be counted in multiple categories if they have more than one diagnosis.

Psychiatric Disorder Categories/Diagnoses	Number of Individuals (%), N = 118
Depressive disorders	76 (64.41)
Anxiety disorders	50 (42.37)
Bipolar and related disorders	40 (33.90)
Substance-related and addictive disorders	22 (18.64)
Seizure disorder	22 (18.64)
Attention-deficit/hyperactivity disorder (ADHD)	22 (18.64)
Trauma- and stressor-related disorders	19 (16.10)
Schizophrenia	11 (9.32)
Personality disorders	10 (8.47)
Borderline personality disorder	10 (8.47)
Disruptive, impulse-control, and conduct disorders	7 (5.93)
Schizoaffective disorder	7 (5.93)
Obsessive-compulsive and related disorder	3 (2.54)
Schizophrenia spectrum and other psychotic disorders	2 (1.69)
Autistic spectrum disorder	2 (1.69)

**Table 3:** Psychiatric Disorder Comorbidities This table summarizes the prevalence of psychiatric disorder comorbidities among the total sample of 138 participants. It categorizes individuals based on the total number of psychiatric diagnoses they carried, ranging from none (0 diagnoses) to four or more diagnoses. The table also presents the average number of revisits associated with each diagnostic category; however, this trend was not statistically significant (Kruskal-Wallis H-Test, p = 0.082).

Number of Psychiatric Diagnoses per Individual	Number of Individuals (%), N = 138	Average Revisits ± Standard Deviation	p-value
0 Diagnosis	20 (14.49)	1.80 ± 0.77	0.082
1 Diagnosis	31 (22.46)	2.10 ± 1.45	-
2-3 Diagnoses	61 (44.20)	2.26 ± 1.68	
4+ Diagnoses	26 (18.84)	2.77 ± 1.42	

The most prevalent medical comorbidities included hypertension (n=28, 20.29% of participants), diabetes (n=15, 10.87%), connective tissue disorders (n=10, 7.25%), chronic obstructive pulmonary disease (n=9, 6.52%), and cancer (n=7, 5.07%). Participants with hypertension tended to have significantly more revisits overall than those without (p = 0.049), as did those with a connective tissue disorder (p = 0.024), and those who suffered a stroke/transient ischemic attack (TIA) (p = 0.016) (Table 4). Reported substance use included tobacco (n=54, 39.13% of participants), alcohol (n=29, 21.01%), and non-alcohol/non-tobacco substances (n=32, 23.19%).

**Table 4:** Medical Comorbidities This table presents the prevalence of medical comorbidities among the total sample of 138 participants. For each comorbidity, the table lists the number and percentage of participants with the diagnosis, along with the mean number of revisits (± standard deviation) for those with and without the diagnosis, and p-values (derived from Mann–Whitney U tests) represent whether there is a statistically significant difference in the distribution of revisit counts between participants with and without each comorbidity. Significant p-values indicated with an asterisk (*). Diagnoses are not mutually exclusive, meaning individuals could be counted in multiple categories if they have more than one diagnosis.

Medical Comorbidities	Number of Individuals (%), N = 138	Average Revisit With Diagnosis ± Standard Deviation	Average Revisit Without Diagnosis ± Standard Deviation	p-value
Hypertension	28 (20.29)	2.79 ± 1.69	2.12 ± 1.42	0.049*
Diabetes dellitus	15 (10.87)	2.93 ± 1.87	2.17 ± 1.43	0.127
Connective tissue disorder	10 (7.25)	3.30 ± 1.89	2.17 ± 1.44	0.024*
Chronic obstructive pulmonary disease	9 (6.52)	2.89 ± 1.96	2.21 ± 1.46	0.338
Cancer	7 (5.07)	1.86 ± 1.07	2.27 ± 1.51	0.566
Liver disease	6 (4.35)	2.83 ± 2.23	2.23 ± 1.46	0.515
Stroke/transient ischemic attack (TIA)	5 (3.62)	4.00 ± 2.00	2.19 ± 1.44	0.016*
Peripheral vascular disease/peripheral arterial disease	3 (2.17)	4.00 ± 2.00	2.21 ± 1.47	0.064
Chronic kidney disease	3 (2.17)	2.33 ± 0.58	2.25 ± 1.51	0.486
HIV	2 (1.45)	1.00 ± 0.00	2.27 ± 1.50	0.124
Congestive heart failure	2 (1.45)	3.50 ± 2.12	2.23 ± 1.49	0.225
Myocardial infarction	2 (1.45)	3.00 ± 1.41	2.24 ± 1.50	0.296
Peptic ulcer disease	2 (1.45)	2.00 ± 0.00	2.26 ± 1.51	0.845

A family history of psychiatric illness was reported in 64 participants (46.38%), with the most common being depressive disorders (n=24, 17.39%) and substance-related and addictive disorders (n=23, 16.67%) (Table 5). Although the average number of revisits was slightly higher among those with a family history (2.31 vs. 2.29), this difference was not statistically significant (p = 0.136). 

**Table 5:** Family History of Psychiatric Disorder Categories/Diagnoses This table presents the prevalence of reported family history of psychiatric conditions among the total sample of 138 participants. It lists the Diagnostic and Statistical Manual of Mental Disorders, Fifth Edition, Text Revision (DSMV-TR) psychiatric disorder categories and specific diagnoses when given in the chart [14]. For each category/condition, the table lists the number and percentage of participants who reported a positive family history. Diagnoses are not mutually exclusive, meaning that individuals could be counted in multiple categories if they reported a family history of more than one psychiatric condition.

Psychiatric Disorder Categories/Diagnoses	Number of Individuals (%), N = 138
Depressive disorders	24 (17.39)
Substance-related and addictive disorders	23 (16.67)
Bipolar and related disorders	15 (10.87)
Anxiety disorders	9 (6.52)
Schizophrenia	4 (2.90)
Trauma- and stressor-related disorders	3 (2.17)
Seizure disorder	3 (2.17)
Attention-deficit/hyperactivity disorder	2 (1.45)
Personality disorders	1 (0.72)
Disruptive, impulse-control, and conduct disorders	1 (0.72)

Event characteristics and stressors 

Between 2019 and 2021, a total of 346 events were recorded, including 311 revisits. Suicide attempts accounted for 268 (77.46%) and self-harm for 78 (22.54%). The mean number of events per participant was 2.51 (SD = 1.50). Among SAs with recorded methods (n = 264), oral medication poisoning was most common (n=145, 54.92%), followed by cutting (n=36, 13.64%) and hanging (n=28, 10.61%). For SH, cutting (n=57, 67.86%) was the most common method, followed by other mechanisms (n=19, 22.62%), and bruising/contusion (n=8, 9.52%). 

Most events (n=251, 72.5%) involved at least one acute stressor, and 123 participants (89.13%) had at least one event with an acute stressor. Common acute stressors included social (168 events, 48.55%), psychological (n=93, 26.88%), and substance-related (n=42, 12.14%). Chronic stressors were reported in 297 events (85.84%), with psychological stressors being the most common (n=263, 76.01%). 

Substance use at the time of the event (not as a method) was documented in 122 events (35.26%), and 64 participants (46.38%) had at least one event associated with substance use. Frequently reported substances included alcohol (61 occurrences, 17.63% of occurrences), cannabis (n=29, 8.38%), cocaine (n=27, 7.80%), heroin (n=25, 7.23%), and benzodiazepines (n=22, 6.36%).

Pre-2019 events and pre-provider visits 

There was a very weak, non-significant correlation between the number of events before 2019 and events from 2019 to 2021 (Spearman’s ρ = 0.107, p = 0.213). While event frequency tended to increase with prior history, the relationship was non-linear and variable. 

Only 43 participants (31.16%) had a documented PCP visit prior to 2019, 8 (5.80%) had seen a psychiatrist, and 3 (2.17%) had seen a therapist or counselor. Those with any pre-2019 outpatient provider visit had a lower, though not statistically significant, revisit rate compared to those without (2.15 vs. 2.31, p = 0.547). 

Risk evaluation, admissions, and disposition 

A Columbia Suicide Severity Rating Scale (C-SSRS) score was documented in 263 events (76.01%); the majority (n=198, 75.30%) received the maximum score of 5. About one-third of events (n=119, 34.39%) resulted in hospital admission. Over half of participants (n=75, 54.35%) had at least one past admission, and the average length of stay was 7.66 days (SD = 11.01). These data are for both patient cohorts who attempted suicide and did self-harm. Among non-admitted events (n = 227), common discharge destinations included inpatient psychiatric or substance use rehabilitation (39.65%), home or group living (38.77%), and correctional facilities (8.81%) (Table 6). Among admitted events (n = 119), common discharge destinations included home or group living facilities (69.75%), inpatient psychiatric or substance use rehabilitation (23.53%), and discharges against medical advice (3.36%) (Table 7). 

**Table 6:** Non-admitted Discharge Dispositions This table summarizes the discharge dispositions categorized for the 227 non-admitted events, with frequencies (n) and percentages calculated relative to the total number of non-admitted events.

Non-Admitted Discharge Dispositions Category	Number of Events (% of 227 total events)
Inpatient psychiatric facility/substance use rehab	90 (39.65)
Home/group living facility (e.g. assisted living)	88 (38.77)
Correctional facility	20 (8.81)
Unknown	20 (8.81)
Other hospital	5 (2.20)
Unhoused	2 (0.88)
Against medical advice	1 (0.44)
Death	1 (0.44)

**Table 7:** Admitted Discharge Dispositions This table summarizes the distribution of discharge dispositions categorized for the 119 admitted events, with event frequencies (n) and percentages calculated relative to the total number of admitted events. Admitted discharge disposition events refer to those events with admission and following discharge from the hospital.

Admitted Discharge Dispositions Category	Number of Events (% of 119 total events)
Home/group living facility (e.g. assisted living)	83 (69.75)
Inpatient psychiatric facility/substance use rehab	28 (23.53)
Against medical advice	4 (3.36)
Correctional facility	2 (1.68)
Other hospital	1 (0.84)
Unknown	1 (0.84)

Outpatient follow-up 

Only 20.23% of events resulted in a scheduled outpatient appointment. Of the 70 scheduled visits, 43 occurred following discharge after a hospital admission. Most scheduled appointments (49, 70.0%) were attended. Primary care follow-up occurred more frequently than psychiatric follow-up (28 vs. 21), with PCP visits more often scheduled within one month, and psychiatric visits within one week of the event (Table 8). 

**Table 8:** Follow-up Visits This table presents the frequency of attended follow-up visits to primary care providers (PCPs) or psychiatrists after discharge following an event. These are displayed by time frame, distinguishing between PCP and psychiatrist follow-ups within defined intervals post-discharge.

Frequency of Time Frame to PCP/Psychiatrist Follow-Up, number (% total follow-up visits of same category)		
Time Frame	PCP	Psychiatrist
Same Day	3 (10.71)	4 (19.05)
1-7 Days	6 (21.43)	12 (57.14)
8-30 Days	11 (30.29)	5 (23.81)
>30 Days	8 (28.57)	0 (0.00)
Total (% of 346 total events)	28 (8.09)	21 (6.07)

## Discussion

Demographics 

Our study did not reveal significant differences in the number of repeat episodes of SA/SH among the various demographic subgroups. Age and ethnicity distribution were similar between our study participants and the county where the data were collected. However, our study was 60.14% female while the county is 50.20% female [10]. This is consistent with previous literature noting a higher percentage of female patients with SA/SH [2]. Another consistent finding is that most individuals included in this study identified as not Hispanic or Latino [2]. Socioeconomic factors, such as financial stress, can increase the risk of suicide attempt [4]. In our study, there was a larger proportion of participants covered by Medicaid, an insurance status used as a proxy for income, although those identified as having middle median household income by zip code were the largest income group (Table 1). 

Participants over 55 years old had the highest average number of revisits. Suicide rates are known to be higher in older adults, highlighting the need for targeted mental health interventions and heightened awareness among clinicians about the risks facing this demographic [15]. On the other hand, the largest age group (40.58%) in our study was 35 to 54 years old, overrepresenting this group that makes up only 24.50% of the local population [10]. The younger age group had the second-highest average number of revisits in our population, which corresponds with data that demonstrates a higher percentage of ED visits for SA/SH among those aged 12 to 17 [2]. This underscores the complexity of evaluating suicide risk across broad age groups and emphasizes the need for clinicians to recognize risk in all age groups. 

Participant history - previous events, stressors 

Previous SA increases the risk of future suicide [6]. Prior SH is also associated with a higher risk of future SH/SA [4]. These relationships are widely accepted, but the magnitude of this risk is disputed. Our study demonstrates a positive, though not statistically significant or linear, relationship between the number of previous SA/SH and future events. 

Most events in our study had acute and chronic stress associated, with social stress being the largest acute stress category and psychological stress being the predominant chronic stressor. Stressors are associated with both SA/SH, a point that offers hope for intervention prior to the event [16]. We advocate for more clinical focus on warning signs of acute or chronic stress as it relates to SH and SA. 

Psychiatric history 

A previous study in Korea showed that the suicide mortality rate among those with psychiatric illnesses is about 5.13-fold higher than that of the general population. Those with bipolar disorder and depressive disorders had the highest suicide mortality rate [17]. Literature also shows a higher psychiatric burden, especially personality disorders like borderline personality disorder, among individuals with SH [18]. 

Compared to the general population, our participants generally experienced higher rates of mental health disorders. A majority of our sample (n=76, 55.07% of the total sample population) had depressive disorders, a markedly higher proportion compared to the 5-17% prevalence in the general population [19]. Additionally, while not statistically significant, the revisits increased with the number of psychiatric diagnoses. This finding aligns with previous research showing a higher prevalence of SAs among individuals with psychiatric comorbidities [20]. Given our findings, clinician suicide screening is imperative for those with psychiatric conditions, especially those with comorbidities. 

Substance use 

Substance use is a well-established risk factor for SAs. A U.S. survey found that individuals with substance dependence were more likely to report a lifetime history of SA compared to non-users (29.5% vs. 19.5%), with non-dependent users showing higher rates (45.3% vs. 19.5%) [21]. Notably, current substance use was a stronger predictor of SA than past use [21]. Supporting this, in our study, nearly half of the participants (46.38%) reported at least one event involving substance use, and 35.26% of total events were associated with substance use. Alcohol was the most frequently reported substance, involved in 18% of events. This highly comorbid relationship reinforces the importance of assessing for SUD risk and diagnosis among all patients and consistent SA/SH screening among patients with a known or probable SUD. 

Family psychiatric history 

Nearly half of the participants (46.38%) reported a family history of psychiatric disorders, most commonly depressive disorders, substance-related and addictive disorders, and bipolar and related disorders. A prior systematic review found that individuals with a family history of mental disorders, suicide, or SA had significantly higher odds of dying by suicide (OR=5.2, OR=3.7, and OR=2.8, respectively) compared to those without [22]. While the precise effect of family history on self-injurious behavior is not fully understood, a meta-analysis suggests a moderate correlation between parental SH and offspring SAs [23]. These findings underscore the importance of assessing family psychiatric history, as it can contribute to a patient's risk for SA/SH. 

Medical history 

Chronic conditions, including hypertension, cancer, COPD, and diabetes, have previously been associated with higher odds of SAs [24]. Similarly, studies have found that patients with repeat SH episodes were more likely to have physical illness and chronic pain [25]. In our study, we found that participants with hypertension, connective tissue disorder, and stroke/TIA had a statistically significantly higher number of SA/SH revisits compared to those without. We believe that hypertension is a key intervention target that may contribute to psychiatric issues. Our study has a small sample size, and further research is needed to explore how medical history, especially hypertension, contributes to SA/SH episodes. 

Method of self-harm/suicide attempt 

Firearm use is the most common and most fatal method of suicide in the United States [26]. However, our study did not capture firearm attempts as those would present to the trauma department, not the ED in the center studied. Consequently, in our study population, poisoning by oral medication was the most common method of suicide attempt, followed by cutting. 

Means restriction is one of the most important suicide prevention strategies [27]. However, reducing access to lethal quantities of medication, including over-the-counter, is challenging. Providers should exercise prudence in prescribing medication that can potentially be used in SA/SH through poisoning. This includes choosing medications with lower associated mortality, such as SSRIs (selective serotonin reuptake inhibitors) over TCA (tricyclic antidepressants) and MAO (monoamine oxidase) inhibitors [28]. Calculating relative lethality can inform dosing and dispensing decisions [29]. Physicians may also limit the quantity of medication dispensed at one time and encourage disposal of excess and expired medication. 

Self-harm methods, without suicidal intent, can be more difficult to reduce access to, such as cutting, which was most common in this study. 

Risk assessment/disposition 

While the C-SSRS is effective in evaluating current suicidal thoughts and behaviors, it has shown limitations in predicting future attempts [30]. Notably, 23.99% of our recorded events lacked a documented C-SSRS assessment, underscoring the need for consistent application during ED visits for SA/SH. 

An analysis of 2016-2020 ED visits for SA/SH in the United States revealed diverse patient dispositions: 24% of visits were admitted to medical, surgical, or psychiatric/detoxification units, 33% discharged, and 30% transferred to psychiatric hospitals [31]. Comparatively, our study found 34.39% of total events resulted in hospital admission, whether medical or psychiatric, 26.01% were transferred to inpatient psychiatric facilities/substance use rehabilitation without admission at the study’s center, and 25.43% ended with discharge to home or a group home without admission. 

Continuity of care 

It is well established that patients recently discharged from a psychiatric unit have a significantly increased suicide risk, especially in the first few months after [32]. Regarding self-harm, a study on repeat episodes after ED discharge found that about a fifth of patients had an SH revisit within a year [33]. A post-discharge follow-up visit after an SA/SH event may prevent future events. One study found that high levels of ambulatory follow-up were significantly associated with lower readmission rates compared to low and medium levels [34]. This demonstrates that continuity of care in an outpatient setting is a critical protective factor. In our study, only a fifth of participants were scheduled for an outpatient follow-up visit, with 70.0% of scheduled encounters attended. There is an urgent role for stronger connections with outpatient resources to reduce SH/SA revisits. 

Continuity of care also remains crucial to managing at-risk patients. In our study, only 31.16% of the sample had pre-2019 PCP visits. Previous PCP visits yielded no statistically significant difference in SH/SA revisits, but on average, those who saw an outpatient clinician before 2019 had 2.15 revisits, lower than the 2.31 revisits for those who did not. Given that a larger percentage of participants saw their PCP prior to our study period and attended more follow-up visits with a PCP compared to other clinicians, this suggests the crucial role PCPs can play in early intervention and the possible prevention of future events. 

Limitations 

In interpreting this study’s findings, limitations warrant consideration. First, reliance on self-reported data introduces recall bias, as individuals may inaccurately remember or report information. Self-reporting may include unsubstantiated events or exclude events for which no medical attention was sought or another hospital unaffiliated with this study’s center was visited. 

Additionally, the use of income level derived from the median income for the zip code, rather than individual income data, may not fully capture socioeconomic disparities and their impact on healthcare utilization. The inability to assess the specific impacts of the COVID-19 pandemic on healthcare-seeking behaviors and outcomes represents a notable limitation, as the pandemic may have influenced care utilization patterns and mental health in ways not fully accounted for in this study. Furthermore, our study reviewed ED revisits, which at the center studied did not capture all cases of self-inflicted firearm and hanging attempts. Our primary hypothesis in this study focuses on identifying risk factors, patterns, and predictors that contributed to these visits. We may need to revisit and possibly conduct multiple locations to capture contextual variations. We have also not included the information on patient visits at other locations outside the study site. We have included patients of various demographics to better represent the target population, but we may still need better representation for future studies. We believe that these will impact the applicability and selection bias of the results. 

## Conclusions

Suicide is a leading cause of death in the United States, and both suicide attempts and self-harm place a significant burden on individuals, communities, and healthcare systems. Understanding the population who revisits for SA/SH along with risk and protective factors informs targeted preventative interventions and care for at-risk individuals. Although this is a retrospective study that focuses on a local community, the findings align with broader trends and provide insights into possible prevention of SA/SH. Addressing the gaps in mental health resources, particularly in follow-up care and means restriction, could possibly reduce repeat SA/SH episodes. Further research is needed to develop comprehensive public health strategies aimed at reducing the burden of suicide and self-harm on individuals and healthcare systems.
